# Elevated maternal non-esterified fatty acid concentrations during late gestation are associated with altered skeletal muscle development and mitochondrial dynamics related-markers in calves

**DOI:** 10.1186/s40104-026-01422-x

**Published:** 2026-06-09

**Authors:** Yu Zhang, Guiling Ma, Xiangyi Zhao, Zhiyong Hu, Yang Gai, Rui He, Shilong Huang, Shengyong Mao, Zhaobing Gu, Yanting Chen

**Affiliations:** 1 College of Animal Science and Technology, Nanjing Agricultural University, Nanjing, 210095 China; 2 College of Agro-Grassland Science, Nanjing Agricultural University, Nanjing, 210095 China; 3 College of Animal Science, Shandong Agricultural University, Taian, 271018 China; 4 Faculty of Animal Science and Technology, Yunnan Agricultural University, Kunming, 650201 China; 5 National Center for International Research On Animal Gut Nutrition, Jiangsu Key Laboratory of Gastrointestinal Nutrition and Animal Health, Nanjing Agricultural University, Nanjing, 210095 China

**Keywords:** Cows, Fetuses, Myogenesis, Non-esterified fatty acids, Skeletal muscle

## Abstract

**Background:**

Late gestation represents an important developmental window for fetal skeletal muscle formation, which is associated with postnatal body growth, metabolic health and mobility in offspring. Although elevated circulating non-esterified fatty acids (NEFA) in dairy cows have been extensively studied in relation to milk production, their associations with fetal and neonatal skeletal muscle development remain unclear.

**Methods:**

Sixty healthy Holstein cows with similar body weight, parity and calving dates were enrolled in this study after dry-off, and retrospectively assigned to low NEFA (*n* = 30, 263 ± 8.8 μmol/L) and high NEFA group (*n* = 30, 379 ± 9.1 μmol/L) according to serum NEFA concentrations at 1, 3, 5, and 7 weeks after dry-off. Skeletal muscle was collected from calves at birth and at one month of age to assess the impacts on offspring muscle mass, fiber morphology and metabolic functions. C2C12 myoblasts were also used to assess the NEFA addition on myogenesis in vitro.

**Results:**

Cows with high NEFA (within the physiological prepartum range) in the dry period had no effect on calf birth weight (*P* = 0.15), but exhibited reduced biceps femoris (*P* = 0.05) and semitendinosus muscle mass (*P* < 0.01). Moreover, calves in the high-NEFA group shifted the muscle fiber composition, characterized by a lower proportion of fast-twitch fibers (*P* < 0.01) and a higher proportion of slow-twitch fibers (*P* = 0.01). The protein abundance of mitochondrial fission-related markers dynamin-related protein 1 (DRP1) and mitochondrial fission protein 1 (FIS1), as well as stimulator of interferon genes (STING)-associated inflammatory markers, was increased in skeletal muscle tissue of calves born to high-NEFA cows (*P* < 0.01). These alterations were persistently observed at one month of age. In vitro, NEFA supplementation reduced myogenic differentiation and fast-twitch myofiber formation (*P* < 0.01), while increasing the expression of mitochondrial dynamics-related genes and inflammatory markers (*P* < 0.01).

**Conclusion:**

Overall, this study reveals that the calves born to mothers with elevated NEFA during the dry period are associated with altered skeletal muscle development and changes in the expression of mitochondrial dynamics- and inflammation-related markers.

**Graphical Abstract:**

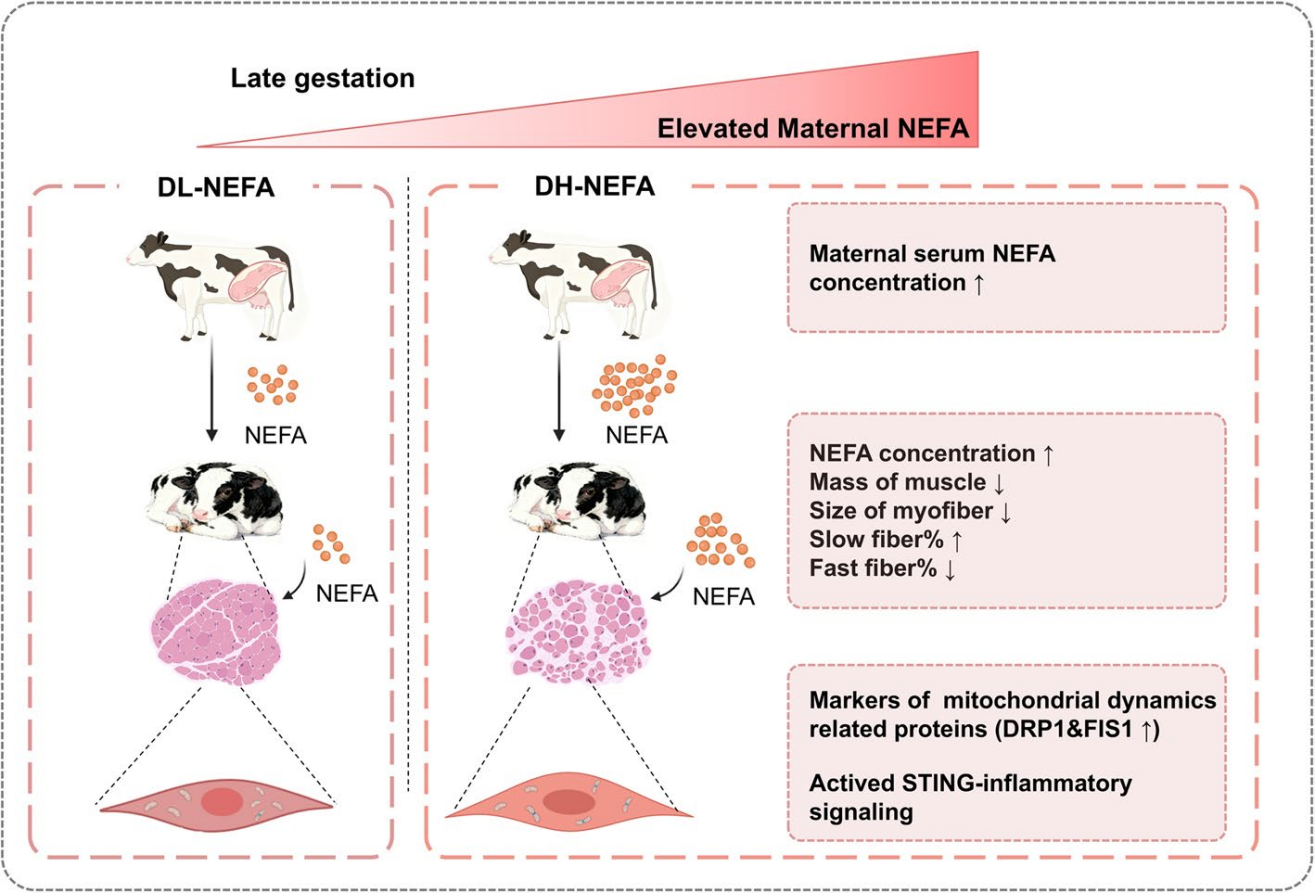

**Supplementary Information:**

The online version contains supplementary material available at 10.1186/s40104-026-01422-x.

## Introduction

During late gestation, fast-growing fetuses and metabolic adaptations for parturition impose significant energy demands on cows. The physiological shifts often lead to negative energy balance (NEB) and body fat mobilization, triggering dramatic increases in serum non-esterified fatty acid (NEFA) concentrations [1]. While NEFA could serve as energy alternative during NEB, the excessive levels beyond homeostatic control (> 0.4 mmol/L for dry-off; > 0.7 mmol/L for early lactation) can predispose cows to metabolic disorders, such as ketosis and fatty liver [2]. Meanwhile, the excessive NEFA in cows could also affect fetal and postnatal growth and metabolic health [3, 4].

Fetal muscle growth is sensitive to maternal metabolic status and nutrition. Maternal malnutrition has been shown to disrupt offspring myogenesis, muscle fiber accretion and alter fiber types [5]. However, the maternal impacts of elevated NEFA concentrations during the dry period on fetal and neonatal muscle development in calves remain poorly understood. The fetal stage, particularly during late gestation, is a critical period for determining the muscle fiber number, types and even metabolic activity in offspring [6]. As skeletal muscles account for 40% of body mass, and control locomotion and energy homeostasis [7], understanding the effects of elevated NEFA during the dry period on fetal muscle growth can be vital in improving animal metabolic health and feed efficiency in dairy management.

Mitochondria are central organelles in regulating energy metabolism, contractile performance and fiber types [8, 9]. High energetic metabolic flux can produce enormous oxidative stress in mitochondria, which relies heavily on the fission process to promptly remove damaged proteins for maintaining mitochondrial quality and metabolic function [8, 10]. However, disruptions of mitochondrial fission, including expressions of dynamin-related protein 1 (DRP1**)** and mitochondrial fission protein 1 **(**FIS1), have been linked to impaired oxidative phosphorylation and mtDNA leakage, inducing significant inflammatory responses, impaired myogenesis [11] and cellular damage [12]. In cows, elevated NEFA concentrations have been associated with mitochondrial dysfunction and cellular apoptosis [13], but the maternal effects during the dry period on mitochondrial fission dynamics in muscle fibers in neonatal calves remain unclear.

Recent evidence suggests that mitochondrial fission could activate stimulator of interferon genes (STING) signaling, which is a key mediator of metabolic stress-induced inflammation [14]. The damaged mitochondrial membrane from an abnormal fission process can activate STING protein, promoting nuclear factor kappa B (NF-κB) cellular inflammation [15]. In rodents, the pathway has been implicated in impaired muscle fiber functions through TANK-binding kinase 1 (TBK1)-mediated NF-κB signaling [16]. However, STING protein and its associated inflammatory regulation in muscle fibers of calves born to maternal high NEFA concentrations also remain unclear.

This study aimed to investigate the association between elevated maternal NEFA concentrations during the dry period and neonatal skeletal muscle development, as well as the expression of genes and proteins related to mitochondrial dynamics and STING-signaling in calves. We hypothesized that elevated maternal NEFA concentrations during the dry period would be associated with altered expression of mitochondrial fission-related genes and proteins and with activation of STING signaling pathways in neonatal skeletal muscle, potentially contributing to changes in muscle fiber growth in calves.

## Methods

### Ethics statement

All experimental procedures in this study were approved by the Ethics Committee on the Use and Care of Animals at Nanjing Agricultural University (#20230625N12; Nanjing, China).

### Animals and experimental design

Sixty healthy Holstein dairy cows were enrolled in this study. Cows were dried off approximately 60 d before the expected calving date using a standardized gradual dry-off protocol. Milking frequency was reduced from twice daily to once daily for 7 d prior to complete cessation of milking. The target dry period duration was 60 d. Before dry-off, fetal sex was determined by transrectal ultrasonography (Mindray DP-3300, Mindray Medical International Ltd., Shenzhen, China). To ensure diagnostic accuracy, sex determination was performed on three separate occasions before dry-off. Only cows consistently identified as carrying male fetuses across all examinations were included in the study. No discrepancies were observed between prenatal sex diagnosis and calf sex at birth. Serum samples were collected from cows via the coccygeal vein [17] at 1, 3, 5, and 7 weeks after dry-off using 10-mL vacuum tubes with a clot activator (Cat. #KW-CN-10, KWS, Shijiazhuang Kangweishi Medical Instrument Co., Ltd., Shijiazhuang, China), and the NEFA concentrations in serum were analyzed by an ELISA-colorimetric test (Cat. #BOFI00145; Assay Genie, Dublin, Ireland). Cows and their corresponding calves were classified into low- and high-NEFA groups based on serum NEFA concentrations measured during the dry period (weeks 1, 3, 5, and 7 after dry-off). For each cow, an individual mean NEFA concentration was calculated by averaging values across the four sampling time points to reflect overall lipid mobilization during the dry period. Cows were subsequently stratified using a median split of these cow-level mean values. Animals with mean NEFA concentrations below the population median (< 323.5 μmol/L) were assigned to the dry period-low NEFA group (DL-NEFA; *n* = 30), whereas those with values at or above the median (≥ 323.5 μmol/L) were assigned to the dry period-high NEFA group (DH-NEFA; *n* = 30). This classification approach was used to capture relative differences in metabolic status within the study population rather than to define established clinical risk categories. All NEFA concentrations remained within physiologically normal ranges, and therefore the DH-NEFA group represented cows with moderately elevated circulating NEFA compared to their herd counterparts. The NEFA concentrations (LSmean ± SEM) were 263 ± 8.8 μmol/L for DL-NEFA and 379 ± 9.1 μmol/L for DH-NEFA. At baseline, no significant differences were observed between DL-NEFA and DH-NEFA cows in body weight (LSmean ± SEM, 742.00 ± 1.20 vs. 739.00 ± 2.20 kg, *P* = 0.53), parity (1.60 ± 0.21 vs. 1.80 ± 0.20; *P* = 0.42), or calving dates (274.90 ± 0.85 vs. 273.70 ± 1.53 d; *P* = 0.51).

Cows were housed in a free-stall barn with dry manure bedding, and had ad libitum access to a total mixed ration (TMR) and clean water. TMR was fed twice daily (0700 and 1700 h) (Table S1). Diet was formulated to meet the nutrient requirements in dry cows following NRC [18]. Body weight was recorded weekly before morning feeding, and feed intake of individuals was recorded weekly. Body condition score (BCS) was evaluated weekly by four independent individuals using a 5-point scoring system [19]. The average of the four scores was calculated and used for statistical analysis. For calves, 6 L pasteurized colostrum was fed by milk bottles to calves immediately after birth. Calves were also fed 6 L of pasteurized whole milk twice daily with free access to water and starters until one month of age. Body weight of calves was determined using a calibrated mobile platform scale (XK3190-A23, Shanghai Yaohua Weighing System Co., Ltd., Shanghai, China). For calves, withers height was measured from ground to scapular apex, and body length was measured from scapulohumeral joint to tuber ischii weekly using a tape meter (100901, Ningbo Great Wall Precision Industrial Co., Ltd., Ningbo, China), following the morphometric protocol [20].

### Dietary nutrient analyses

The TMR provided to dry cows underwent weekly sampling, with dry matter (DM) content determination performed through forced-air oven drying at 55 °C. Processed samples were homogenized to pass a 1-mm mesh sieve (Wiley mill, model 4; Arthur H. Thomas Co., Philadelphia, PA, USA) following oven drying. Chemical analyses included determination of crude protein (CP, method 984.13), ether-extracted (954.02), acid detergent fiber (ADF, 973.18), ash (942.05), and mineral composition following Association of Official Analytical Chemists (AOAC) [21] procedures. Neutral detergent fiber (NDF) and starch quantification were determined by sequential detergent system following the previous procedure [22].

### Skeletal muscle collection and processing

Fifteen neonatal calves from each group (*n* = 15) were randomly selected and euthanized by bolt stunning immediately after birth [23]. An additional fifteen calves per group were fed until one month of age, and euthanized by bolt stunning for skeletal muscle collections, including biceps femoris, semitendinosus, and rectus femoris. Muscles were individually collected, weighed and frozen in −80 °C freezer. Taking as a large portion of muscle depots, biceps femoris was further fixed in 4% paraformaldehyde (Cat. #G1101; Servicebio, Wuhan, China) at 4 °C for following histological analyses.

### Serum sample collection and analysis

Serum in cows was collected from coccygeal vein at 0600 h before morning feeding at 1, 3, 5, and 7 weeks after dry-off using 10 mL vacuum tubes coated with clot activator (Cat. #KW-CN-10, KWS, Shijiazhuang Kangweishi Medical Instrument Co., Ltd., Shijiazhuang, China). Before colostrum feeding, serum was also drawn in individual calves from the jugular vein. Samples were allowed to clot at room temperature (RT) 30 min before centrifugation at 1,500 × *g* for 15 min at 4 °C, and serum was stored at −20 °C for further analysis. β-Hydroxybutyrate (BHB, Cat. #MM-5100301, Jiangsu Meimian Industrial Co., Ltd., Yancheng, China) [24], insulin (Cat. #RD-RX77142, Shanghai Henghui Biotechnology Co., Ltd., Shanghai, China) [25] and NEFA (Cat. #BOFI00145; Assay Genie, Dublin, Ireland) [26] in serum were quantified using ELISA kits following the manufacturers’ protocols, respectively. Serum glucose was measured using the test strips (Cat. #07124112, Roche Diagnostics GmbH, Mannheim, Germany) [27].

### Hematoxylin and eosin (H&E) staining

Muscle fiber cross-sectional areas were analyzed by H&E staining [28, 29]. Briefly, biceps femoris was fixed in 4% paraformaldehyde at 4 °C for 24 h, following paraffin-embedding and 5 μm thickness sectioning in a microtome (HM 355 S; SLEE medical GmbH, Nieder-Olm, Germany). Paraffin-embedded sections were dewaxed in two stages of xylene for 5 min each followed by a series of graded ethanol dehydration. Hematoxylin staining was performed for 1 min, after which the sections were rinsed under running water for 2 min. Eosin staining was then applied for 1 min. Subsequently, the stained sections underwent dehydration in absolute ethanol, and clearing in xylene to prepare for microscopic examination. Muscle fiber cross-sectional area was quantified from six images per section at 400 × magnification (210–330 muscle fibers per slide), and images were analyzed by ImageJ (NIH, Bethesda, MD, USA; version 1.54g). For nuclei counting on slides using ImageJ, color deconvolution was initially applied to isolate staining components, followed by conversion to 8-bit grayscale with optimized brightness/contrast. Particle separation was achieved through sequential binary thresholding and watershed segmentation, before automated quantification was applied via Analyze Particles. Size determination was utilized to scale images with a 50-μm reference line, with fiber dimensions subsequently measured using the same analytical module.

### In vitro NEFA supplementation and myogenetic differentiation

C2C12 myoblasts (CL-0044; Wuhan Pricella Biotechnology Co., Ltd., Wuhan, China) were seeded in 6-well plates (2.5 × 10^5^ cells/mL) and cultured in low-glucose Dulbecco's Modified Eagle Medium (DMEM; Cat. #C11330500BT; Gibco, Thermo Fisher Scientific, Waltham, MA, USA), with 10% fetal bovine serum (FBS, Cat. #U11–059A; YOBIBIO (Shanghai) Biotechnology Co., Ltd., Shanghai, China), 100 U/mL penicillin and 1,000 U/mL streptomycin (Cat. #15140122; Gibco, Thermo Fisher Scientific, Waltham, MA, USA). Cells were cultured in a 5% CO_2_ incubator (Wci-260; Wiggens Deutschland GmbH, Heidelberg, Germany) at 37 °C. Upon myoblasts reaching 80% confluency, the growth medium was replaced with myogenic differentiation medium including DMEM with 2% horse serum (Cat. #BL209A; Biosharp, Hefei, China), 2% bovine serum albumin (Cat. #9048-46-8; Boer, Shanghai, China), and 1% antibiotics according to the previous studies [30–32]. For induction, the myogenic differentiation lasted 6 d to form mature myofibers, and the culture medium was replaced once per two days [33]. Before myogenic induction, myoblasts appeared as small, short-length phenotypes under light microscopy. Upon substitution with 2% horse serum, the myoblasts elongated and fused to form myofibers, as observed under light microscopy. Immunostaining with Desmin and DAPI was performed to assess fiber formation, and the expression of muscle heavy chains was quantified using qPCR to further validate the maturation.

During the differentiation, myoblasts were subjected to three treatments, including control (PBS; 0 μmol/L NEFA), 100 μmol/L NEFA, or 200 μmol/L NEFA as described previously [34, 35]. Each treatment was applied to three technical replicate-wells per experiment, and the entire experiment was independently repeated three times (*n* = 3 biological replicates). NEFA was prepared as a stock solution (52.7 mmol/L, pH 7.4) as previously described [36, 37]. Briefly, NEFA stocking solution, which includes oleic acid (22.9 mmol/L; Cat. #SC9320, Servicebio, Wuhan, China), linoleic acid (2.6 mmol/L; Cat. #SL8520, Servicebio, Wuhan, China), palmitic acid (16.8 mmol/L; Cat. #SP8060, Solarbio, Beijing, China), stearic acid (7.6 mmol/L; Cat. #SS8520, Servicebio, Wuhan, China), and palmitoleic acid (2.8 mmol/L; Cat. #P794587, Shanghai Macklin Biochemical Technology Co., Ltd., Shanghai, China), was dissolved in 0.1 mol/L KOH and further heated to 60 °C, followed by pH adjustment to 7.4 using 1 mol/L HCl. After 6 days of differentiation, muscle fibers were stained, and imaged by a confocal fluorescence microscopy (Leica DMi8, Leica Microsystems, Wetzlar, Germany) [38]. Mature fibers were also harvested for mRNA and protein analyses.

### Immunofluorescence analysis

Paraffin-embedded muscle sections were deparaffinized by immersion in two stages of xylene exchange followed by dehydration through graded ethanol solutions. Antigen retrieval was performed by incubating the sections in 1 mmol/L EDTA buffer (pH 8.0) at 95 °C for 20 min, with the container covered to prevent buffer evaporation. After cooling and three 5-min PBS washing, endogenous peroxidase activity was quenched with 3% H_2_O_2_ at 25 °C for 15 min. Sections were blocked with 5% donkey serum (Cat. #BL1051A; Biosharp, Hefei, China) and 1% BSA (Cat. #B607846; Shanghai Boer Biotechnology Co., Ltd., Shanghai, China) for 1 h to block non-specifics, followed by overnight incubation with primary antibodies against myosin heavy chain 1 (MYH1, 1:200; Cat. #GB112130-50; Servicebio, Wuhan, China) or myosin heavy chain 7 (MYH7, 1:300; Cat. #GB111857; Servicebio, Wuhan, China) at 4 °C. For muscle fibers in vitro, cells were washed with cold PBS, fixed with 4% paraformaldehyde for 15 min, and permeabilized with 0.2% Triton X-100 (Cat. #P0096; Beyotime, Shanghai, China) for 10 min, following by blocking with 3% BSA (Cat. #B607846; Shanghai Boer Biotechnology Co., Ltd., Shanghai, China) for 30 min. Fibers were incubated with an anti-Desmin primary antibody (1:200; Cat. #A0699, ABclonal, Wuhan, China) overnight at 4 °C. Secondary antibody staining was performed under light protection using Cyanine 3 (Cy3)-conjugated goat anti-rabbit IgG (1:1,000; Cat. #AS007, ABclonal, Wuhan, China) for Desmin and MYH7, or fluorescein isothiocyanate-conjugated goat anti-rabbit IgG (1:1,000; Cat. #AS011, ABclonal, Wuhan, China) for MYH1. Nuclei were co-stained by 4',6-Diamidino-2-phenylindole (DAPI, 5μg/mL; Cat. #RM02978, ABclonal, Wuhan, China). Samples were mounted with anti-fade medium (Cat. #S2100; Solarbio, Beijing, China), and imaged using a STELLARIS confocal microscope (Leica Microsystems, Wetzlar, Germany; fluorescein isothiocyanate: 488/519 nm, Cy3: 552/570 nm, DAPI: 358/461 nm). Areas of different muscle fiber types were measured in six fields per section at 200 × magnification using ImageJ (NIH, Bethesda, MD, USA; v. 1.54g). The proportion of each muscle fiber type was calculated as: (area of fiber type X/total muscle fiber area) × 100% [39]. Fluorescence quantification was performed using ImageJ (NIH, Bethesda, MD, USA; v. 1.54g) with background-normalized intensity measurements following previous studies [40, 41]. Background-subtracted fluorescence intensity was measured from at least 3 randomly selected fields per sample. The fluorescence intensity of the control group (0 μmol/L NEFA) was arbitrarily set to 1, and relative intensities of other groups were expressed as fold change.

### Real-time qPCR analysis

Total RNA in tissues or cells was extracted using TRIzol reagent (Cat. # 15596026CN, Thermo Fisher Scientific, Carlsbad, CA, USA) following instructions and previous studies [41, 42]. RNA quality and concentration were assessed using a NanoDrop 2000 (Thermo Fisher Scientific, Waltham, MA, USA). Total RNA (1,000 ng) was reverse transcribed using an iScript cDNA synthesis kit (Cat. #TSK302S, Tsingke Biotechnology Co., Ltd., Beijing, China) following the manufacturer’s protocol. The thermal cycling included 42 °C for 2 min, 60 °C for 5 min, 55 °C for 15 min, and final enzyme inactivation at 85 °C for 5 min. mRNA expression was quantified using SYBR Green Supermix (Cat. #TSE201; Tsingke Biotechnology Co., Ltd., Beijing, China) on a QuantStudio™ 7 Flex Real-Time PCR System (Thermo Fisher Scientific, Waltham, MA, USA), with raw cycle threshold values processed using QuantStudio™ Design and Analysis Software v1.4 (Thermo Fisher Scientific, Waltham, MA, USA). mRNA expression was normalized to reference genes including 18S rRNA and glyceraldehyde-3-phosphate dehydrogenase (GAPDH) as reported in previous studies [40, 43], and the expressions were normalized via the 2^−ΔΔCt^ method [43, 44]. Primer sequences were validated and synthesized by Tsingke Biotech (Beijing, China, Table S2).

### Western blot analysis

Proteins in tissue or cells were extracted in biceps femoris or cells using 1 mL of ice-cold RIPA lysis buffer (Cat. #BL504A; Biosharp, Hefei, China) with protease inhibitors (Cat. #BL507A; Biosharp, Hefei, China). Protein concentration was quantified via a bicinchoninic acid assay kit (Cat. #P0012; Beyotime, Shanghai, China). Approximately 15 µg proteins were loaded, and separated by SDS-PAGE electrophoresis (Cat. #89,888; Thermo Fisher Scientific, Waltham, MA, USA), and transferred onto polyvinylidene fluoride membranes (Cat. #IPVH10100; MilliporeSigma, Burlington, MA, USA). Membranes were blocked with 1.5% skim milk for 2 h at RT, and further incubated overnight at 4 °C with primary antibodies against DRP1 (1:200; Cat. #A21968, ABclonal, Wuhan, China), FIS1 (1:500; Cat. #A5821, ABclonal, Wuhan, China), STING (1:500; Cat. #A21051, ABclonal, Wuhan, China), TBK1 (1:1,000; Cat. #A3458, ABclonal, Wuhan, China), or β-tubulin (1:1,000; Cat. #AF2835, Beyotime, Shanghai, China), respectively. Following incubation with HRP-conjugated secondary antibodies, including horseradish peroxidase-conjugated goat anti-rabbit IgG (H + L) (1:1,000; Cat. #A0208, Beyotime, Shanghai, China) or horseradish peroxidase-conjugated goat anti-mouse IgG (H + L) (1:1,000; Cat. #A0216, Beyotime, Shanghai, China) for 2 h at RT, protein bands were detected by an enhanced chemiluminescence (Cat. #RM00021P, ABclonal, Wuhan, China). Bands were imaged on a ChemiDoc MP system (Bio-Rad, Hercules, CA, USA), and the intensities were quantified by Protein Simple Systems (ProteinSimple, San Jose, CA, USA). Protein contents were normalized to β-tubulin as an internal control.

### Statistical analysis

An a priori power analysis was conducted using the *simr* package in R to determine the minimum number of animals required for the maternal NEFA treatment experiment. Based on effect sizes estimated from our previous NEFA data and the planned mixed-effects model structure, a minimum of 10 cows per group was required to detect a biologically meaningful difference with 80% statistical power at a significance level of α = 0.05. Statistical analysis was performed by *lme4* (v.1.1–35) package in R 4.5.2 [45], and animal experiment units were cows or calves. Residual normality was checked by Shapiro–Wilk method in *stats* package in R. Variance homogeneity was estimated using Levene’s test and visualized using quantile–quantile plots [46]. After checking the variable normality, no log transformations were needed in the analyses. An ANOVA mixed model was chosen to analyze dry cow metabolic and body condition indexes, including serum metabolites, nutritional intake, body weight, BCS, and serum parameters, with treatment (maternal NEFA levels), time, their interaction as fixed effects, and cow as a random effect. For variables without time effects in this study, data were analyzed using an ANOVA mixed model, with treatment as the fixed effect. Data from calves at birth or one-month-old were analyzed separately (*n* = 15). Bonferroni’s method was used to adjust the confidence intervals. Heterogeneous autoregressive type 1 structure was selected for data variance–covariance structure estimation based on Akaike and Bayesian criteria. Results are presented as least-squares means (LSmean) ± SEM, where SEM represents the standard error of the LSmean estimated from the mixed-effects model rather than the raw standard deviation. *P* < 0.05 is considered as a significant difference, 0.05 ≤ *P* < 0.10 is considered as tendency.

## Results

### Body conditions in dry cows and birth weight in calves

**Fig. 1:**
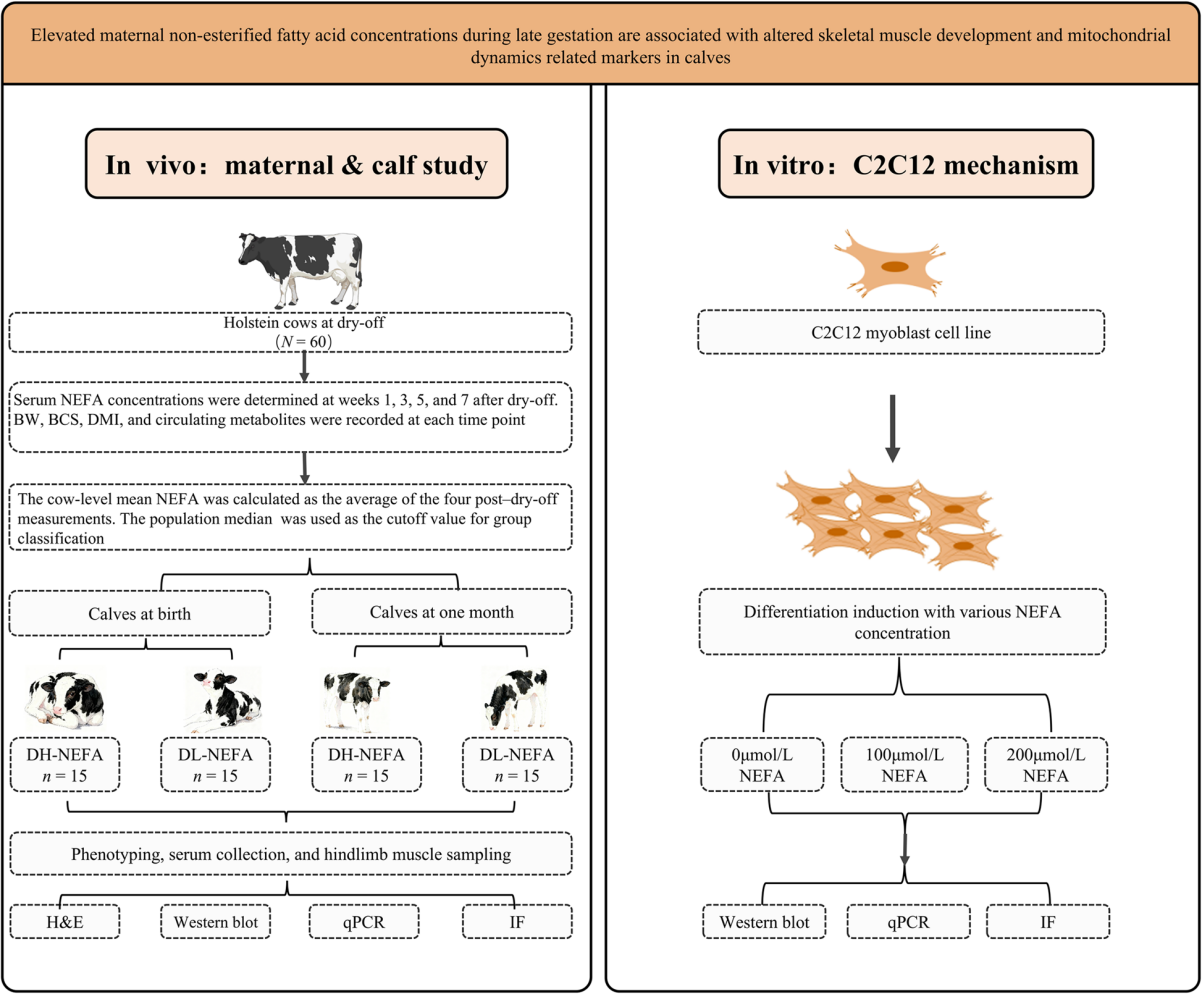
Schematic overview of the experimental design. In vivo, 60 Holstein cows were enrolled at dry-off. Serum non-esterified fatty acid (NEFA) concentrations were measured at weeks 1, 3, 5, and 7 after dry-off, together with body weight (BW), body condition score (BCS), dry matter intake (DMI), and serum metabolites including β-hydroxybutyrate (BHB), glucose, and insulin. Cows were classified into low-NEFA (DL-NEFA; *n* = 30; NEFA < 323.5 μmol/L) and high-NEFA (DH-NEFA; *n* = 30; NEFA ≥ 323.5 μmol/L) groups using a median split based on each cow’s mean NEFA concentration across the four sampling weeks. Male calves (*n* = 15 per group) were sampled at birth and at 30 days of age for phenotyping, serum collection, and hindlimb muscle sampling. Muscle tissues were subjected to hematoxylin and eosin (H&E) staining, immunofluorescence (IF), qPCR, and Western blot analyses. In vitro, C2C12 myoblasts were induced to differentiate and treated with NEFA (0, 100, or 200 μmol/L), followed by IF, qPCR, and Western blot analyses

**Fig. 2:**
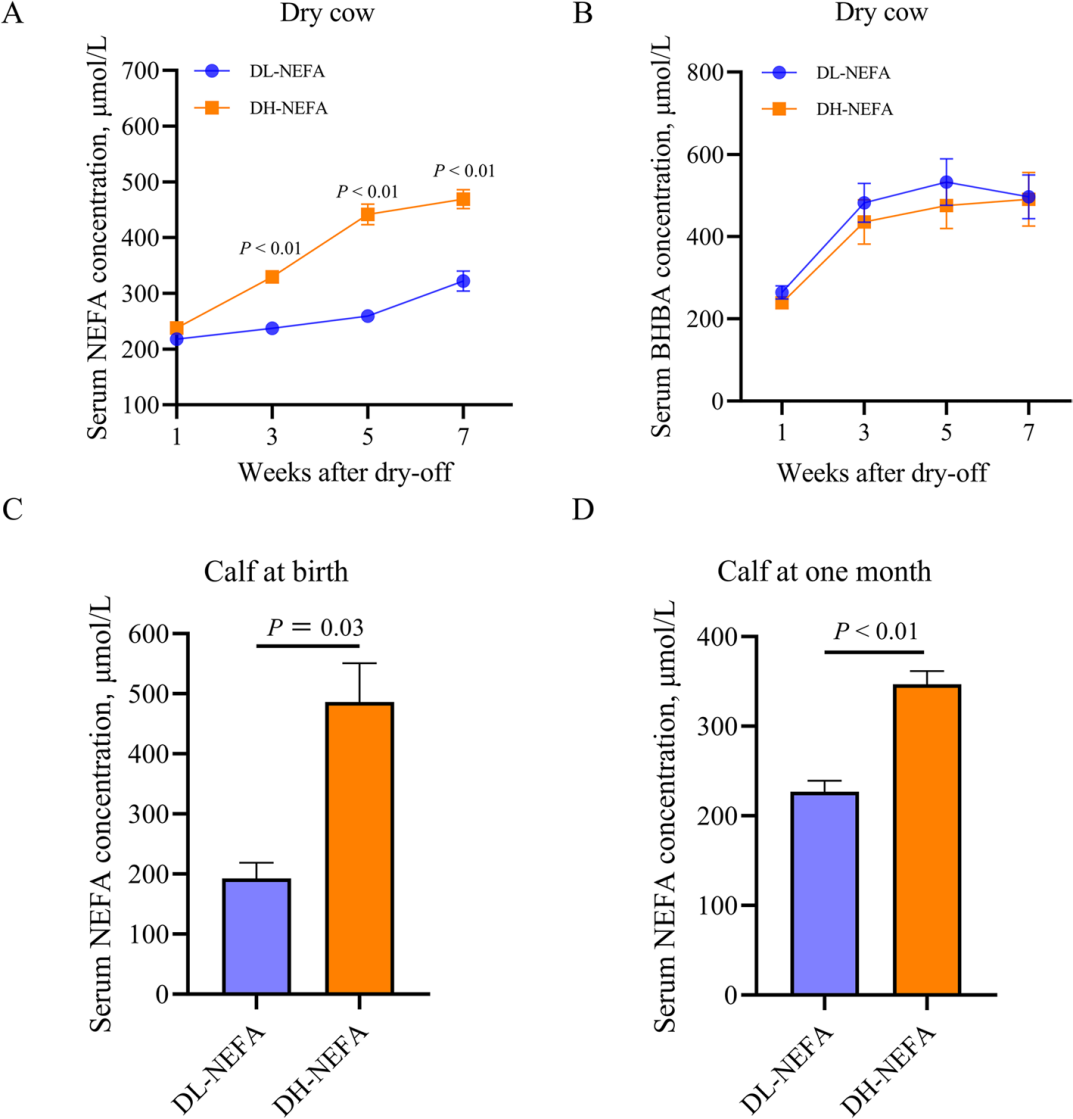
Concentrations of non-esterified fatty acids (NEFA) and β-hydroxybutyrate (BHB) in dry cows and calves at birth and at one month.** A** Serum NEFA concentrations in cows from DL-NEFA and DH-NEFA groups after dry-off. **B** Serum BHB concentrations in dry cows. **C** Serum NEFA concentrations in calves at birth. **D** Serum NEFA concentrations in calves at one month of age. DL-NEFA, dry cows with low serum NEFA concentration; DH-NEFA, dry cows with high serum NEFA concentration. Data are presented as LSmean ± SEM (*n* = 30 per group)

The overall experimental workflow, including maternal grouping, calf sampling time points, and in vitro validation, is summarized in Fig. 1. Cows in the DH-NEFA group exhibited persistently higher serum NEFA concentrations during the dry period (treatment effect, *P* < 0.001; Fig. 2A; *n* = 30 per group, 379 ± 9.1 μmol/L vs. 263 ± 8.8 μmol/L, *P* < 0.01). A significant effect of week (*P* < 0.001) and a treatment × week interaction (*P* < 0.001) was also detected. Weekly measurements demonstrated that this difference was consistently maintained at 3, 5, and 7 weeks after dry-off, indicating a persistent elevation rather than a transient fluctuation. Importantly, NEFA concentrations in both groups remained within ranges reported for clinically healthy prepartum cows. Serum concentrations of BHB, glucose, and insulin remained comparable between groups (Fig. 2A and B and Table 1). For BHB, there was no treatment effect (*P* = 0.539) and no treatment × week interaction (*P* = 0.781), whereas a significant week effect was observed (*P* < 0.001; Fig. 2B). The absence of differences in BHB is consistent with the prepartum physiological state and supports that elevated NEFA in DH-NEFA cows was not accompanied by overt ketogenesis. However, NEFA concentrations alone do not comprehensively reflect maternal metabolic status, which may also be influenced by additional circulating metabolites and inflammatory indicators.

**Table 1:** Maternal serum concentrations and body conditions of dry cows in this study

Variable^b^	Treatment^a^		SEM	*P*-value		
	DL-NEFA	DH-NEFA		NEFA	Week	NEFA × Week
Glucose, mmol/L	4.13	4.23	0.061	0.08	0.42	0.63
Insulin, μIU/mL	7.01	6.9	0.13	0.60	0.91	0.67
DMI, kg/d	13.1	13.4	0.45	0.72	0.51	0.76
BW, kg	746	742	7.64	0.53	0.02	0.87
BCS	3.3	3.3	0.06	0.82	0.90	0.62

^a^*DL-NEFA* Dry cows with low NEFA in serum (*n* = 30), *DH-NEFA* Dry cows with high NEFA in serum (*n* = 30)

^b^*NEFA* Non-esterified fatty acids, *DMI* Dry matter intake, *BW* Body weight, *BCS* Body condition score

Values are presented as least squares means (LSmean) ± SEM (*n* = 30 cows per group)

**Fig. 3:**
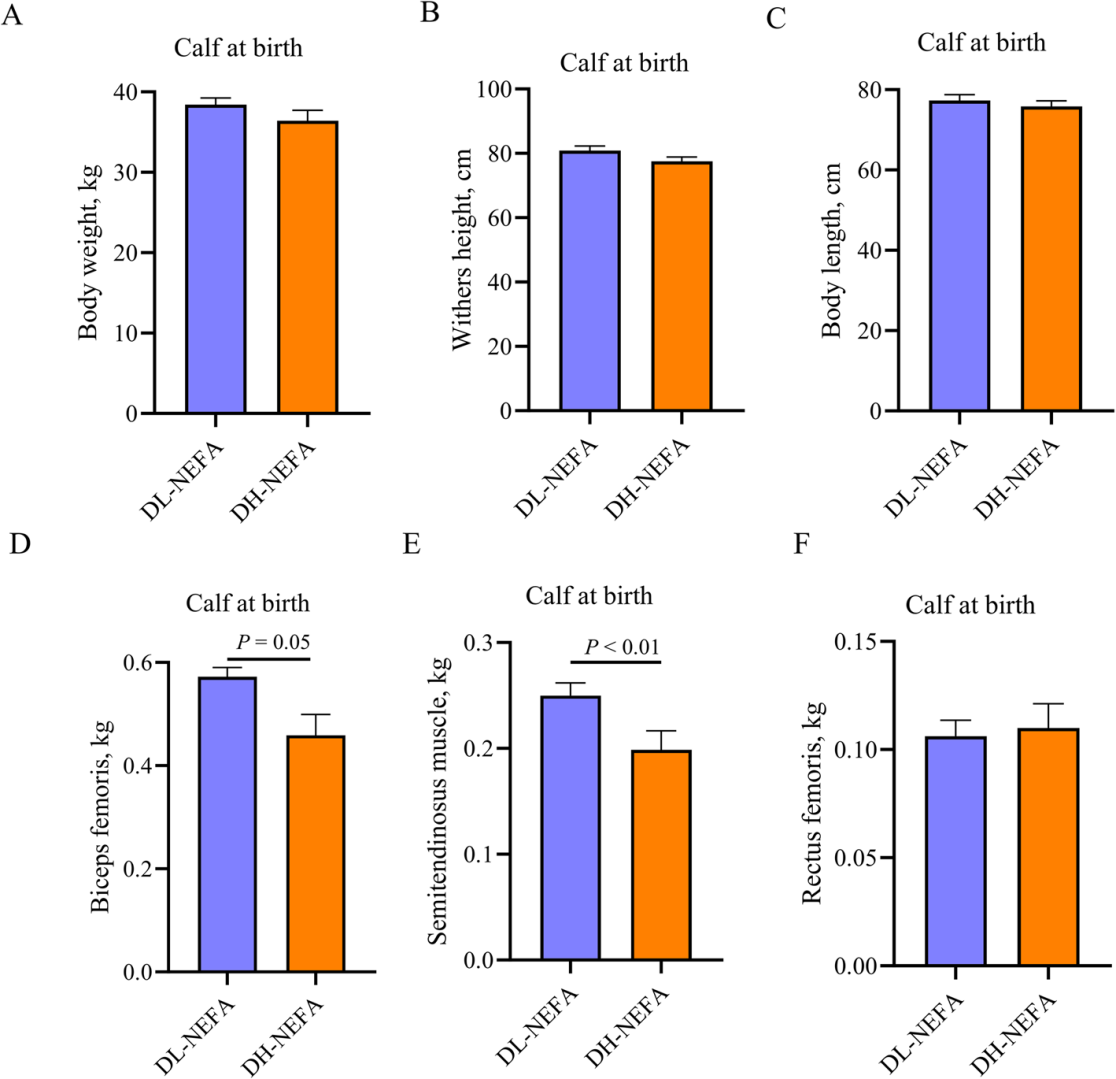
Effects of maternal non-esterified fatty acid (NEFA) levels on body growth and skeletal muscle mass in calves at birth. **A** Body weight. **B** Withers height. **C** Body length. **D** Tissue mass of biceps femoris. **E** Semitendinosus. **F** Rectus femoris muscles. DL-NEFA, dry cows with low serum NEFA concentration; DH-NEFA, dry cows with high serum NEFA concentration. Data are presented as LSmean ± SEM (*n* = 15 per group)

Dry matter intake (DMI) was similar between DL-NEFA and DH-NEFA cows during the dry period (13.1 vs. 13.4 kg/d, SEM = 0.45; *P* = 0.72; Table 1) and remained stable across the four sampling weeks (week effect; *P* = 0.51). Likewise, body weight (746 vs. 742 kg, SEM = 7.64; *P* = 0.53) and BCS (3.3 vs. 3.3, SEM = 0.06; *P* = 0.82) did not differ between groups and remained stable throughout the experimental period. These results indicate that cows were metabolically comparable in terms of intake and body reserves, with circulating NEFA being the primary differing metabolic parameter. Consistent with maternal profiles, serum NEFA concentrations in calves at birth in DH-NEFA group were higher than calves in DL-NEFA (*P* = 0.03, Fig. 2C), which were also persistent in calves at one month of age (*P* < 0.01; Fig. 2D). However, for calves at birth, body weight (*P* = 0.15), withers height (*P* = 0.12) and body length (*P* = 0.27) were similar between groups (Fig. 3A–C).

### Skeletal muscle development and mitochondrial dynamics-related molecular alterations in calves at birth

**Fig. 4:**
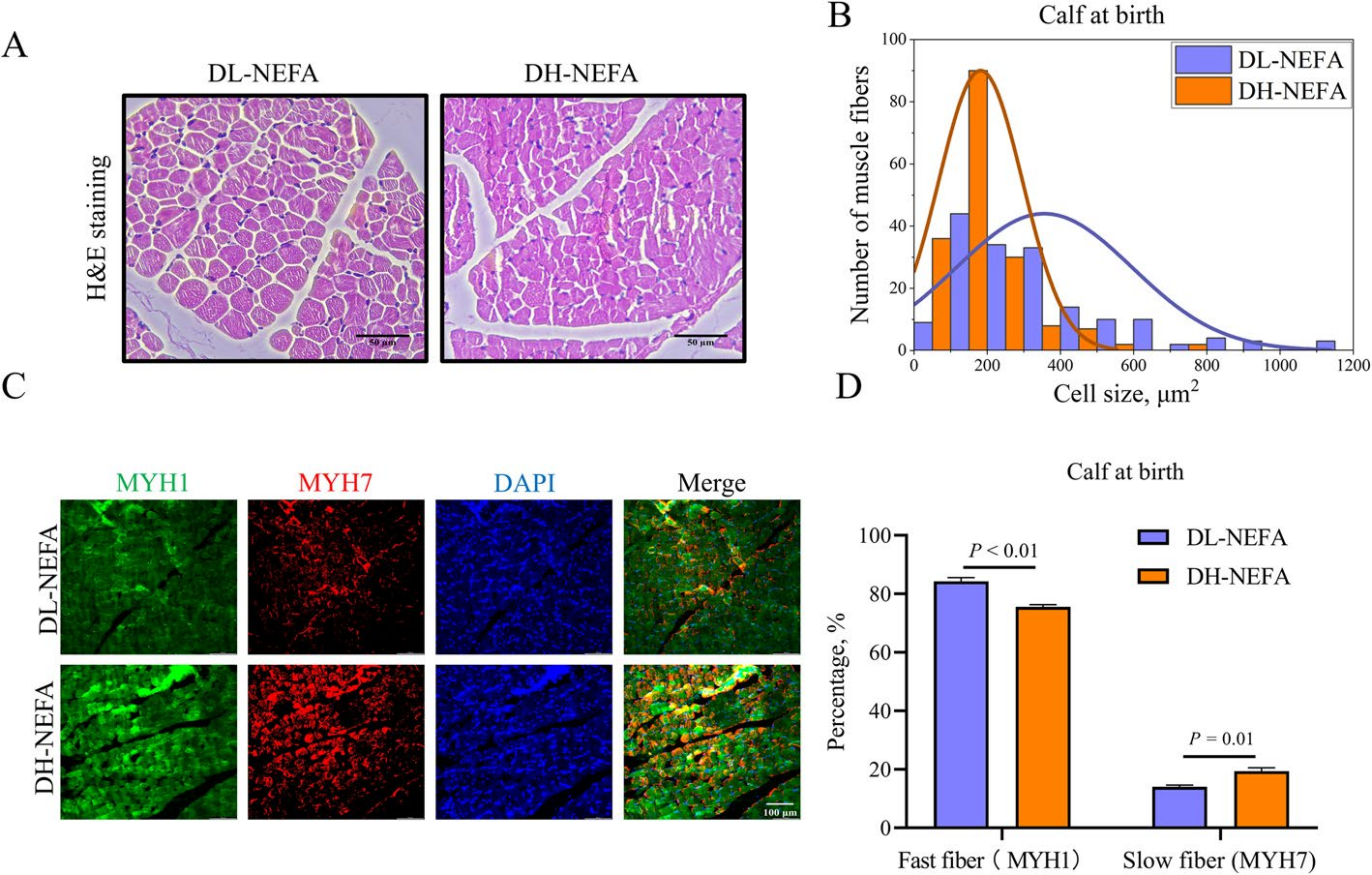
Effects of maternal non-esterified fatty acid (NEFA) levels on biceps femoris muscle morphology and fiber-type composition in newborn calves. **A** Hematoxylin and eosin (H&E) staining of biceps femoris muscle and quantification of muscle fiber size (scale bar = 50 μm). **B** Quantification of muscle fiber cross-sectional area. **C** Immunofluorescence staining of fast-twitch (MYH1, green) and slow-twitch (MYH7, red) fibers with DAPI nuclear counterstaining (scale bar = 100 μm). **D** Quantification of fast- and slow-twitch fiber proportions. DL-NEFA, dry cows with low serum NEFA concentration; DH-NEFA, dry cows with high serum NEFA concentration. Data are presented as LSmean ± SEM (*n* = 8 per group)

**Fig. 5:**
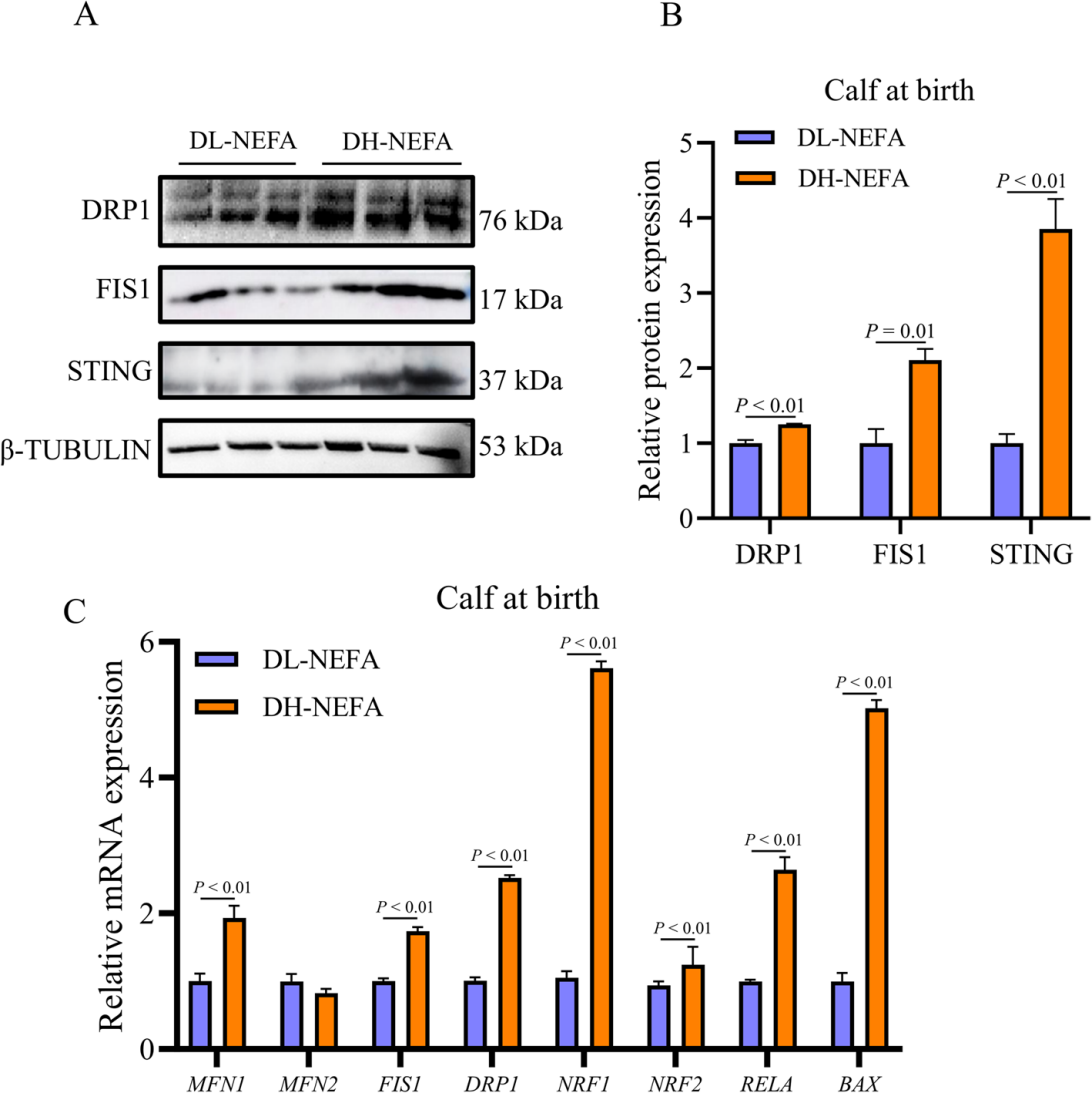
Effects of maternal non-esterified fatty acid (NEFA) levels on mitochondrial dynamics and related protein expression in the biceps femoris muscle of newborn calves. **A** and** B** Western blot analysis of mitochondrial fission-related proteins (DRP1, FIS1) and STING. Protein abundance was normalized to β-tubulin. **C** Relative mRNA expression of mitochondrial dynamics-related genes in biceps femoris muscle. DL-NEFA, dry cows with low serum NEFA concentration; DH-NEFA, dry cows with high serum NEFA concentration. For relative expression analysis, DL-NEFA group was set to 1, and values in the DH-NEFA are expressed relative to the DL-NEFA group. Data are presented as LSmean ± SEM (*n* = 8 per group)

Despite similar body condition indexes at birth, calves from DH-NEFA exhibited significantly lower mass of biceps femoris (*P* = 0.05) and semitendinosus muscles (*P* < 0.01) compared with DL-NEFA calves (Fig. 3D–F). Notably, the mass of rectus femoris was not altered (*P* = 0.99), showing the tissue-depot dependent effects. To determine the effects on morphology and gene expressions in muscle fibers, the biceps femoris muscles were further used to examine the details. Cross-sectional analysis revealed decreases in muscle fiber size in DH-NEFA calves (Fig. 4A and B). Myosin heavy chain 1 or 7 immunostaining further uncovered 9.45% less fast-twitch (*P* < 0.01; Fig. 4C and D) but 5.35% more slow-twitch fibers in biceps in DH-NEFA calves (*P* = 0.01; Fig. 4C and D). In biceps, the contents of mitochondrial fission proteins, including DRP1 and FIS1, were significantly elevated in DH-NEFA calves relative to DL-NEFA calves (Fig. 5A and B). Similarly, mRNA expression of mitochondrial dynamic-regulatory genes, including *MFN1*, *FIS1*, *DRP1*, *NRF1*, *NRF2* and *BAX*, were also upregulated in DH-NEFA calves relative to DL-NEFA calves (*P* < 0.01; Fig. 5C). Consistent with increased abundance of fission-related markers, STING protein and mRNA expression of the inflammatory gene *RELA*, which encodes the p65 subunit of the immune-related transcription factor NF-κB, were significantly increased in DH-NEFA calves (*P* < 0.01; Fig. 5C), suggesting alterations in mitochondrial dynamics-related pathways and inflammatory signaling in skeletal muscle.

### Muscle fiber growth and mitochondrial dynamics-related molecular alterations in one-month-old calves

**Fig. 6:**
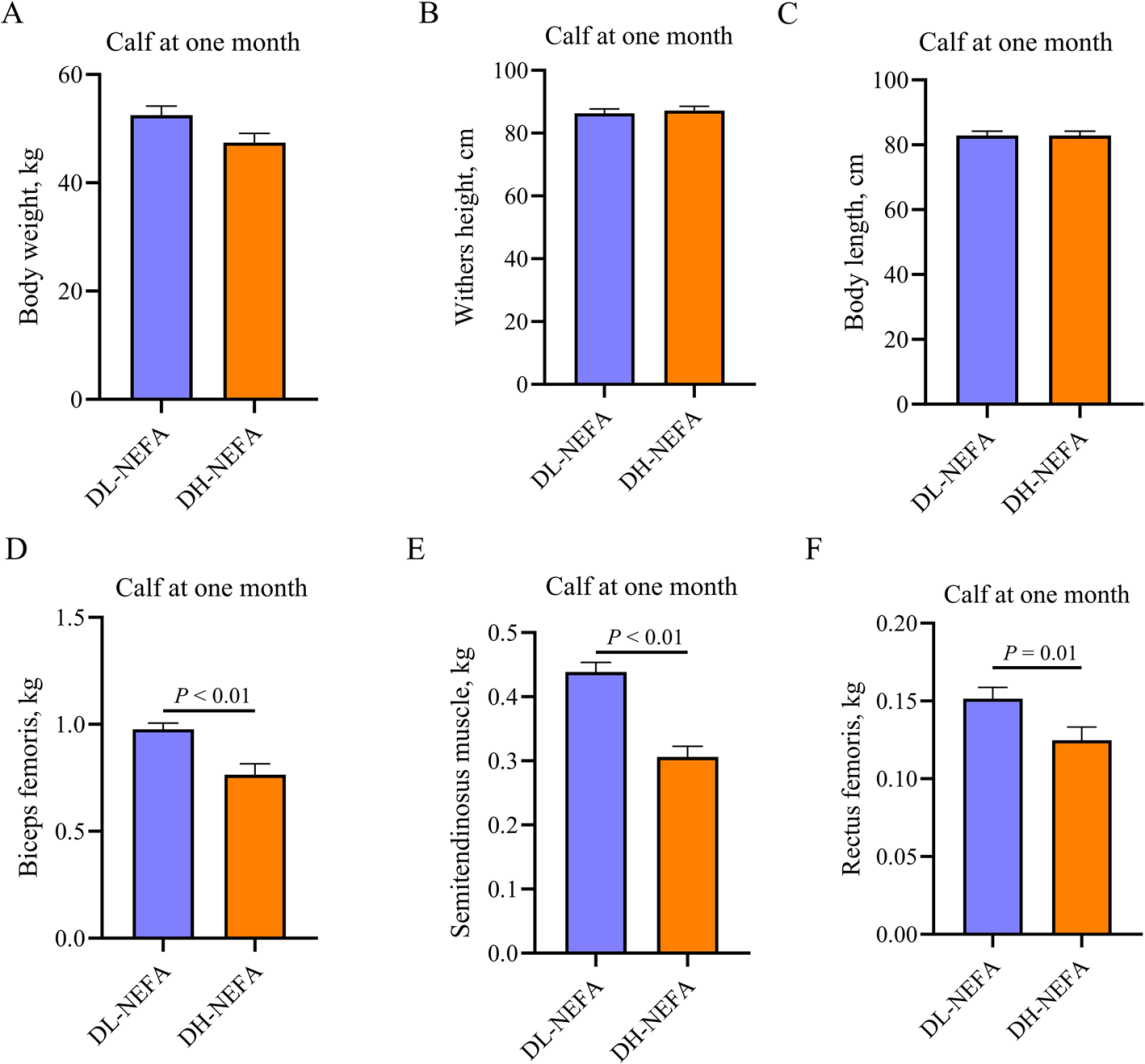
Effects of maternal non-esterified fatty acid (NEFA) levels on growth performance and muscle mass in one-month-old calves. **A** Body weight. **B** Withers height. **C** Body length. **D** Mass of biceps femoris. **E** Semitendinosus. **F** Rectus femoris muscles. DL-NEFA, dry cows with low serum NEFA concentration; DH-NEFA, dry cows with high serum NEFA concentration. Data are expressed as LSmean ± SEM (*n* = 15 per group)

**Fig. 7:**
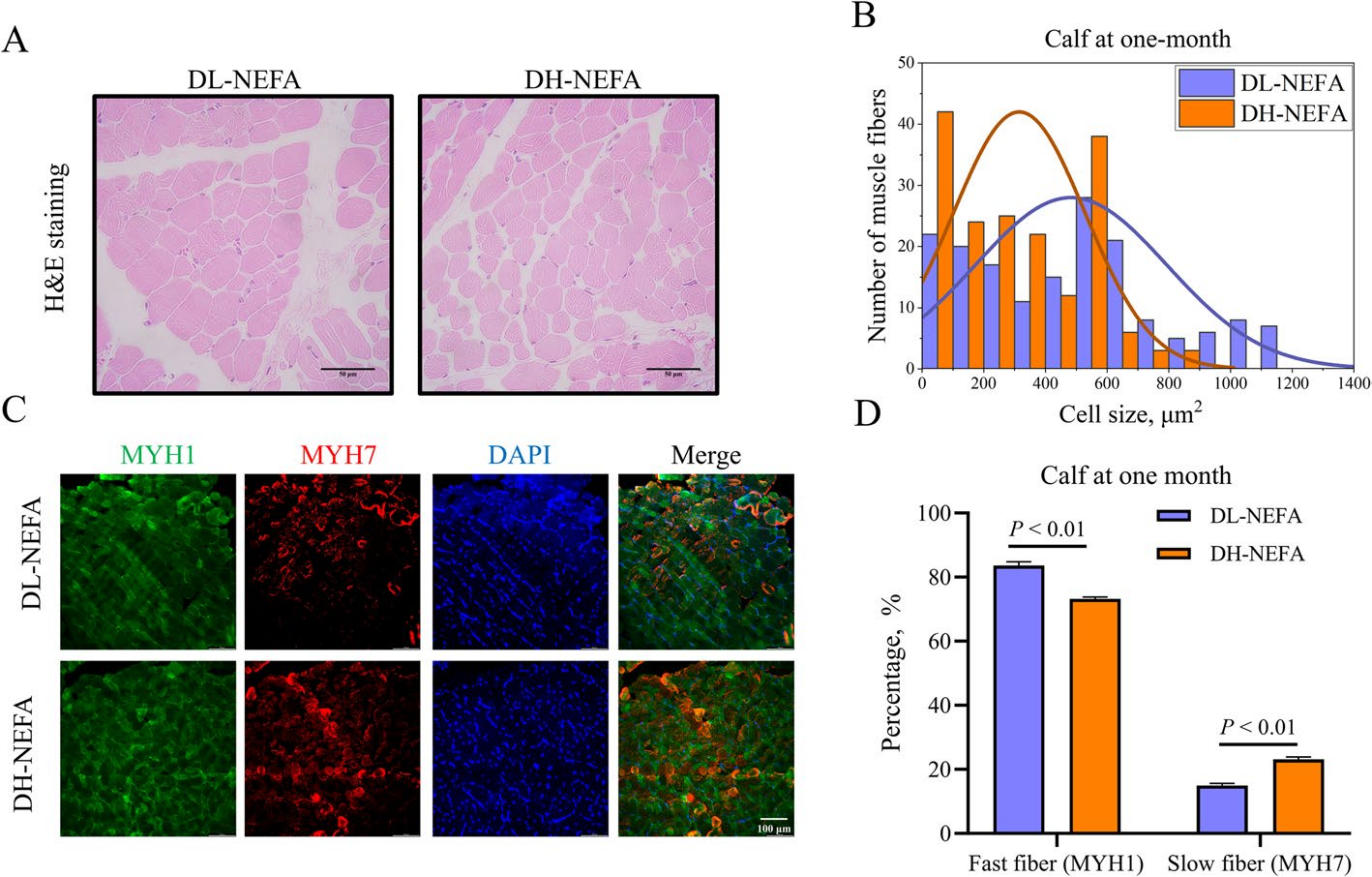
Effects of maternal non-esterified fatty acid (NEFA) levels on skeletal muscle morphology and fiber-type composition in one-month-old calves. **A** Hematoxylin and eosin (H&E) staining of biceps femoris muscle (scale bar = 50 μm). **B** Quantification of muscle fiber cross-sectional area. **C** Immunofluorescence staining of fast-twitch (MYH1, green) and slow-twitch (MYH7, red) fibers with DAPI nuclear counterstaining (scale bar = 100 μm). **D** Quantification of fast- and slow-fiber composition; DL-NEFA, dry cows with low serum NEFA concentration; DH-NEFA, dry cows with high serum NEFA concentration. Data are expressed as LSmean ± SEM (*n* = 8 calves per group)

To determine the postnatal impacts on muscle growth, calves born to DL-NEFA and DH-NEFA cows were also monitored until one month of age. No significant difference observed in body weight (*P* = 0.1; Fig. 6A), withers height (*P* = 0.66; Fig. 6B) and body length (*P* = 0.97; Fig. 6C) in calves at one month. However, the weights of biceps femoris (*P* < 0.01; Fig. 6D), semitendinosus (*P* < 0.01; Fig. 6E) and rectus femoris muscles (*P* < 0.01; Fig. 6F) were all lower in DH-NEFA group, suggesting an existence of persistent and aggregated deficit in postnatal muscle growth. Furthermore, cross-sectional analysis of the biceps confirmed 10.4% smaller muscle fiber size (*P* < 0.01; Fig. 7A and B), and sustained increases of slow-twitch fibers (*P* < 0.01; Fig. 7C and D) in DH-NEFA group.

**Fig. 8:**
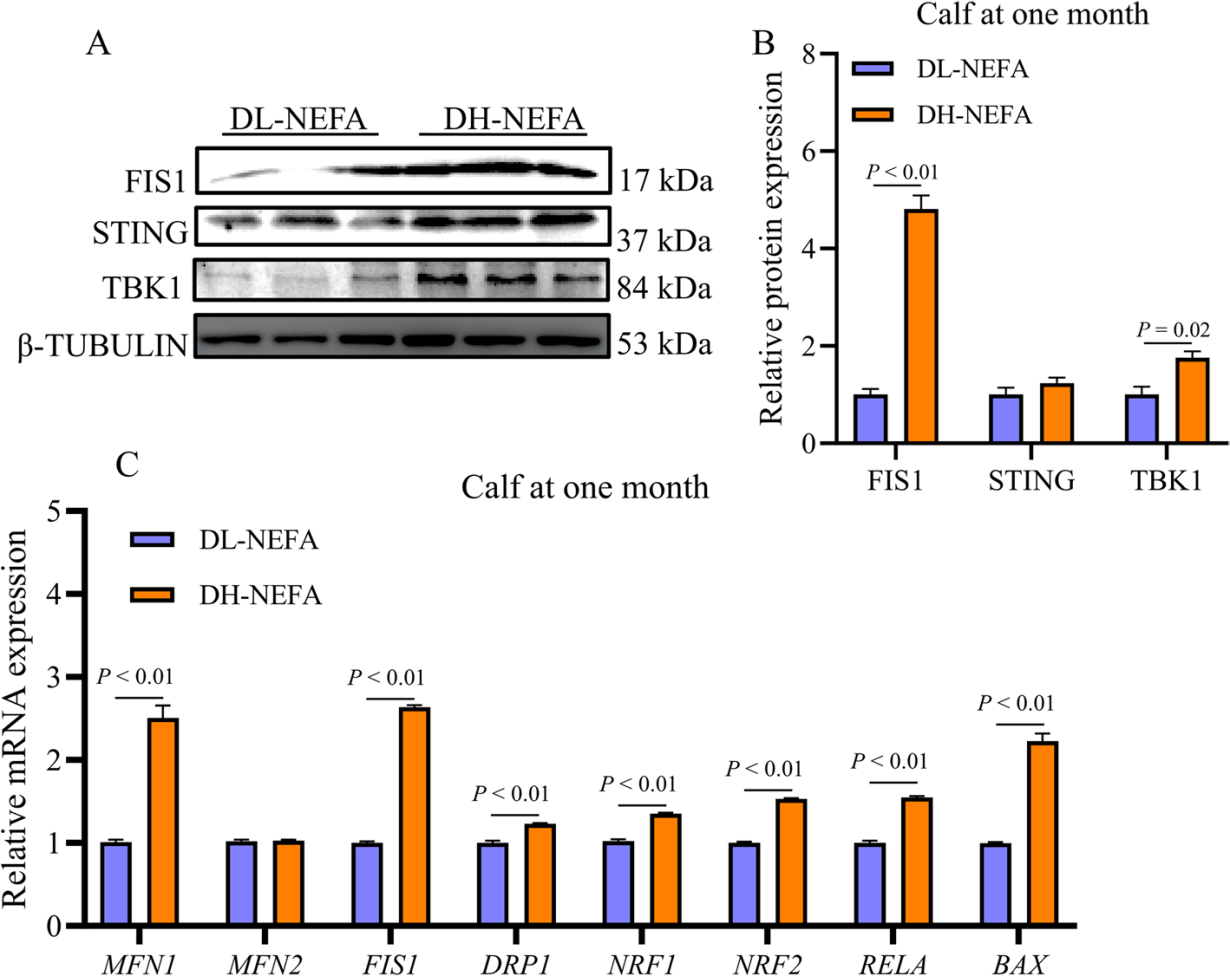
Effects of maternal non-esterified fatty acid (NEFA) levels on mitochondrial dynamics and related protein expression in the skeletal muscle of one-month-old calves. **A** and** B** Western blot analysis of mitochondrial fission-related proteins (DRP1, FIS1) and STING. Protein abundance was normalized to β-tubulin. **C** Relative mRNA expression of mitochondrial dynamics-related genes in biceps femoris muscle. DL-NEFA, dry cows with low serum NEFA concentration; DH-NEFA, dry cows with high serum NEFA concentration. For relative expression analysis, DL-NEFA group was set to 1, and values in the DH-NEFA are expressed relative to the DL-NEFA group. Data are presented as LSmean ± SEM (*n* = 8 calves per group)

In biceps, DH-NEFA calves also exhibited significant upregulations of mitochondrial dynamics-related genes, including *MFN1, FIS1*, *DRP1*, *NRF1*, *NRF2* and *BAX* (*P* < 0.01; Fig. 8C). Inflammatory gene *RELA* was also highly activated in DH-NEFA calves (*P* < 0.01; Fig. 8C). Protein analysis displayed an increased protein content of FIS1 (*P* < 0.01) and TBK1 (*P* = 0.02) in DH-NEFA, while STING protein levels remained unchanged (*P* = 0.26, Fig. 8A and B). These observations suggest that alterations in mitochondrial dynamics- and inflammation-related markers persisted postnatally.

### Effects of NEFA supplementation on myogenic differentiation and mitochondrial dynamics-related gene expression in vitro

**Fig. 9:**
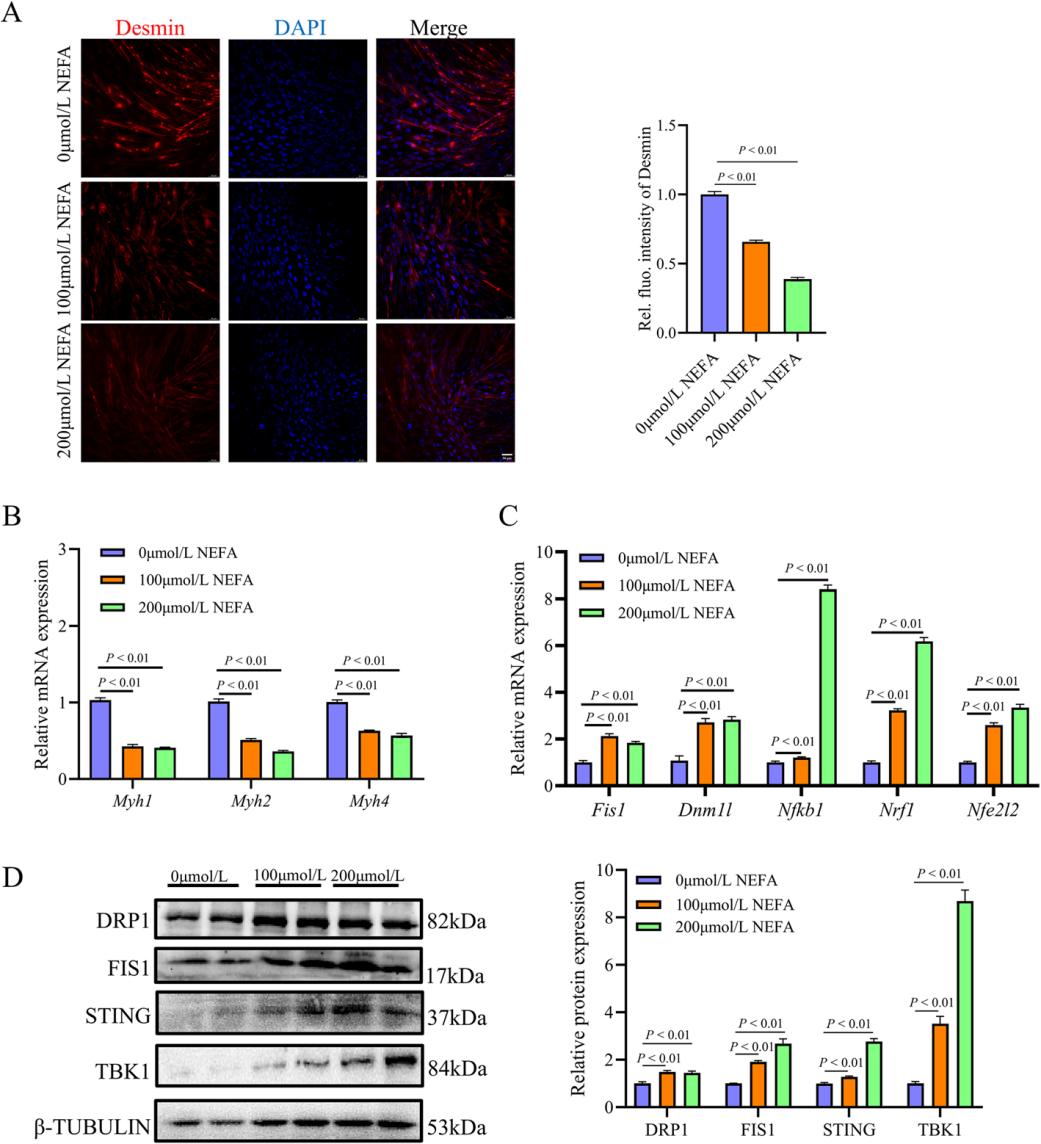
Effects of dosed non-esterified fatty acids (NEFA) treatment on myogenic differentiation and mitochondrial dynamics in C2C12 cells. **A** Immunofluorescence staining and relative fold changes of Desmin intensity with DAPI nuclear counterstaining (scale bar = 50 μm).** B** The mRNA expression of fast-twitch fiber formation-associated genes. **C** The mRNA expression of mitochondrial dynamics- and STING-signaling-related genes. **D** Western blot analysis of DRP1, FIS1, STING and TBK1. Protein abundance was normalized to β-tubulin. The 0 μmol/L NEFA group was set to 1, and treatment groups were expressed relative to the 0 μmol/L NEFA group. Data are presented as LSmean ± SEM (*n* = 3 independent experiments; each treatment performed in triplicate)

To further validate the impacts of high NEFA on muscle fiber formation, C2C12 myoblasts were subjected to the dosed NEFA supplementation during the myogenic differentiation in vitro. After 6-day myogenic induction, Desmin protein immunostaining revealed significant decreases in mature fiber formations in either low or high dose of NEFA addition relative to controls (*P* < 0.01, Fig. 9A). Expressions of fast-twitch fiber-regulatory genes, including *Myh1*, *Myh2*, and *Myh4*, were highly downregulated (*P* < 0.01, Fig. 9B), while mitochondrial fission genes, including *Fis1*, *Dnm1l*, *Nrf1*, and *Nfe2l2 *(*P* < 0.01; Fig. 9C), were upregulated by NEFA addition. Moreover, the mRNA expression of *Nfkb1* inflammatory regulator was also elevated (*P* < 0.01), aligned with the elevations of DRP1, FIS1, STING and TBK1 protein contents (*P* < 0.01) (Fig. 9C and D), indicating that NEFA exposure was associated with reduced myogenic differentiation and increased expression of mitochondrial fission-related and inflammatory markers.

## Discussion

Adequate energy supplies during the dry period are critical for fetal growth and development. Maternal energy deficits during pregnancy often lead to alterations in prenatal and postnatal calf growth and metabolic health [47]. Elevated NEFA acts as a metabolic stressor in cows during pregnancy, which can affect the placental functions and nutrient supplies to the fetus, and also increase the risks of abnormal offspring growth after birth [48]. In particular, the unsaturated long-chain fatty acids have high abilities to cross the placenta, which may expose the fetus to maternal NEFA influences. Although extensive studies have assessed the negative impacts of high serum NEFA concentrations on metabolic health in cows [49], and a recent study showed the serum NEFA levels above 300 μmol/L during the last two weeks before calving were highly associated with the low birth weight [4], the impacts on fetal and postnatal muscle growth, morphology, and functions in calves remain largely unknown. In this study, we observed that NEFA levels in the DH-NEFA group (LSmean: 379 μmol/L) remained below the subclinical ketosis threshold (NEFA < 400 μmol/L) during the dry period and fell within the normal metabolic range for healthy dry cows, rather than indicating pronounced lipomobilization or overt negative energy balance. It is therefore important to acknowledge that the observed programming effects on offspring muscle development occurred under mild maternal metabolic variation rather than metabolic disease. This distinction does not diminish the scientific value of these findings; rather, it reinforces the novelty of the observation that even subtle differences in maternal lipid status within the physiologically normal range may be sufficient to affect neonatal muscle biology. Consistent with this interpretation, calves born to DH-NEFA cows showed no significant differences in whole-body growth indices at birth [50], and similar growth metrics including body weight, body length and withers height were also observed at one month of age. Interestingly, we observed that calves born to DH-NEFA cows exhibited impaired muscle growth in various depots, and also altered mitochondrial fission and STING-related immune responses. Furthermore, NEFA addition consistently limited the myofiber formation, and also promoted shifts in fiber type toward slow-twitch fibers, highlighting that maternal high NEFA during the dry period may be linked to the impaired muscle development and function, and high risks of lameness and low milk performance in next generation cows. Given the limited transfer of long-chain saturated fatty acids across the placenta, polyunsaturated fatty acids could be major components of maternal NEFA associated with the physiologic alterations in skeletal muscles of calves.

Elevated maternal NEFA levels during the dry period have been linked to impaired immune functions in offspring, as evidenced by the reduced cytokine production under stress [51]. Driven by the dramatic nutrient demands for fetal growth in late gestation, body fat mobilization in cows increases the serum NEFA concentrations due to NEB [52]. Besides, the reduced DMI can also exacerbate this situation and further contribute to increased serum NEFA concentrations in cows. In this study, DH-NEFA calves at birth and one month of age showed no differences in body weight, body length, or withers height compared with those in low NEFA group, which may be attributed to NEFA levels remaining below the ketosis threshold [50, 53], suggesting that the NEFA elevation below the ketosis threshold may not be severe enough to induce the alterations in whole-body growth metrics, but can sufficiently affect skeletal muscle growth, morphology, and functions. Aligned with the recent study showing a positive correlation between maternal serum NEFA concentration and NEFA levels in calves within 24 h after birth [54], we also observed that calves born to dams with high NEFA had significantly higher serum NEFA at birth, supporting that maternal NEFA may play a crucial role in affecting fetal tissue or organ development.

Late gestation is a critical period for fetal muscle growth [6], since muscle fibers are mainly developed and matured during this period. Fetal muscle growth is highly sensitive to maternal energy status, as undernutrition during pregnancy can alter developmental programming of myofibers and satellite cell proliferation, leading to long-term consequences in whole-body growth after birth [6]. While the negative impacts of high serum NEFA concentration on cow health have been well studied previously, including the impaired liver functions, metabolic health and reproduction [55], in this study, we further highlighted the negative associations of maternal high-NEFA with impaired muscle growth, morphology, and functions in calves.

Skeletal muscle plays pivotal roles in animal mobility, protein body reserves, immune functions and feed efficiency [7, 56]. In the current study, calves born to maternal high-NEFA cows exhibited significantly reduced muscle fiber size, indicating altered muscle morphological development, which may have implications for subsequent muscle growth capacity [6, 57]. Given that MYH1 protein is crucial for rapid muscle growth and contraction, the protein suppression may contribute to the reduced muscle development in calves born to high NEFA cows [58].

Mitochondrial dynamics are essential for muscle growth and function [59, 60]. In this study, calves in the DH-NEFA group exhibited increased expression of mitochondrial fission-related genes *FIS1* and *DRP1*, which may indicate increased mitochondrial stress signaling [61]. Notably, the expression of MFN1, a key regulator of mitochondrial fusion, was also significantly upregulated. Mitochondrial fusion and fission are highly coordinated and interdependent processes that together regulate mitochondrial quality control and bioenergetic adaptation [10, 62]. Therefore, the concurrent upregulation of both fission- and fusion-related genes suggests a dynamic remodeling of the mitochondrial network rather than a simple shift toward fragmentation. Such coordinated regulation has been reported under conditions of metabolic or oxidative stress, where enhanced mitochondrial turnover and structural reorganization are required to maintain cellular homeostasis [8, 10]. As the mitochondrial fission processes are typically enhanced under mild stress to facilitate removal of damaged mitochondrial components and optimize energy production [10], the observed upregulation of stress-related genes, *NRF1*, *NRF2*, *RELA* and *BAX*, further supports the presence of altered mitochondrial dynamics and metabolic stress in the skeletal muscle of calves born to high NEFA cows [63].

Beyond serving as lean mass, skeletal muscle also plays an active role in immune regulations, with STING signaling as a key mediator in mitochondrial stress [14]. In this study, STING protein abundance was increased in DH-NEFA calves, indicating that elevated maternal NEFA is associated with enhanced inflammatory signaling in skeletal muscle. The observations are consistent with the previous study demonstrating that excessive or dysregulated inflammatory responses in skeletal muscle, characterized by persistent activation of innate immune pathways, increased pro-inflammatory cytokine production, and impaired macrophage phenotypic transition-can disturb the balance between muscle degeneration and regeneration. Such immune imbalance has been shown to impair myofiber repair capacity, promote mitochondrial dysfunction, and ultimately contribute to muscle weakness and delayed regeneration [64]. To further investigate the direct effects of high NEFA concentration on myogenesis and mitochondrial dynamics, we conducted in vitro studies and also consistently observed the high NEFA addition can suppress myoblast differentiation, and upregulate mitochondrial fission and inflammatory genes [65], supporting a potential relationship between maternal NEFA levels and offspring muscle molecular profiles.

Overall, the strength of this study lies in the combined in vivo and in vitro approaches to illustrate the relationship between maternal high NEFA during the dry period and skeletal muscle growth in calves at birth and one month of age. Although we did not observe the significant alterations in calf body weight or body length, the offspring from the high NEFA group exhibited lower skeletal muscle mass and metabolic activity. Moreover, the shifts in muscle fiber types and mitochondria-associated STING signaling were also uncovered as potential mediators of those maternal effects, underscoring the importance of metabolic health and energy homeostasis of pregnant dry cows for optimal offspring muscle development and functions.

However, it should be acknowledged that maternal circulating NEFA concentration alone does not fully characterize the overall metabolic status of dry cows. While NEFA is widely recognized as an indicator of lipid mobilization and energy balance, other metabolic and physiological factors including glucose and insulin dynamics, hepatic function, inflammatory mediators, amino acid availability, and fatty acid composition-may also influence the intrauterine environment and contribute to offspring developmental outcomes.

Due to the technical limitations, this study was designed to examine associations between maternal circulating NEFA concentrations and changes in offspring skeletal muscle growth and molecular profiles. Therefore, causal relationships cannot be inferred from the present data. Further studies are needed to determine direct effects and clarify the underlying mechanisms. Furthermore, additional factors such as glucose and amino acid deficiencies may also contribute to the observed alterations, and other signaling pathways beyond STING may be involved in regulating muscle fiber growth. For example, the mammalian target of rapamycin pathway, which regulates muscle growth and metabolism, may contribute to the NEFA-induced muscle alterations [66]. Additionally, the Hippo signaling pathway and microRNAs also potentially play crucial roles in post-transcriptional gene regulation responsive to maternal metabolic stress [67]. Future research could further explore these pathways to elucidate the maternal and fetal reprogramming effects in dairy management. It should be noted that mitochondrial function was not directly assessed in this study; therefore, conclusions regarding mitochondrial functional impairment are based on changes in the expression of mitochondrial dynamics-related genes and proteins.

## Conclusion

In summary, our study showed that calves born to dry cows with elevated NEFA exhibited no differences in body weight; however, they displayed significant alterations in skeletal muscle development and molecular characteristics. These included shifts in muscle fiber type composition, increased expression of mitochondrial fission-related genes and proteins, and elevated STING-associated inflammatory markers. The results emphasized that the sufficient energy supply and metabolic health during the dry period are critical for prenatal and postnatal healthy growth in calves.

## Supplementary Information

Additional file 1: Table S1. Ingredient and chemical composition of diets fed to dry cows.

Additional file 2: Table S2. Primer sequences used in qPCR analyses.

Additional file 3. The original Western blot images

## Data Availability

All data analyzed in this study are included in the tables and figures of this manuscript.

## Abbreviations

BAX: BCL-2-associated X protein
BCS: Body condition score
BHB: β-Hydroxybutyrate
DAPI: 4',6-Diamidino-2-phenylindole
DH-NEFA: Dry cows with high serum NEFA concentration
DL-NEFA: Dry cows with low serum NEFA concentration
DM: Dry matter
DMI: Dry matter intake
DRP1: Dynamin-related protein 1
FIS1: Mitochondrial fission protein 1
GAPDH: Glyceraldehyde-3-phosphate dehydrogenase
H&E: Hematoxylin and eosin
MFN1: Mitofusin 1
MFN2: Mitofusin 2
MYH1: Myosin heavy chain 1
Myh2: Myosin heavy chain 2
Myh4: Myosin heavy chain 4
MYH7: Myosin heavy chain 7
NEB: Negative energy balance
NEFA: Non-esterified fatty acids
NF-κB: Nuclear factor kappa B
Nfkb1: Nuclear factor kappa B subunit 1
NRF1: Nuclear respiratory factor 1
NRF2: Nuclear factor erythroid 2-related factor 2
RELA: V-rel avian reticuloendotheliosis viral oncogene homolog A (p65)
STING: Stimulator of interferon genes
TBK1: TANK-binding kinase 1
