# Supplementary material for: Elevated maternal non-esterified fatty acid concentrations during late gestation are associated with altered skeletal muscle development and mitochondrial dynamics related-markers in calves
Source: J Anim Sci Biotechnol. 2026 Jun 9;17:114. doi: 10.1186/s40104-026-01422-x (PMC13248329; doi:10.1186/s40104-026-01422-x)
Supplement: Supplementary file 1 — Additional file 1: Table S1. Ingredient and chemical composition of diets fed to dry cows. [file 40104_2026_1422_MOESM1_ESM.docx]

**Table S1** Ingredient and chemical composition of diets fed to dry cows

| **Item** | **Diet** |
| --- | --- |
| Ingredients, % DM |  |
| Corn silage | 44.4 |
| Barley | 11.1 |
| Wheat straw | 16.7 |
| DDGS^1^ | 16.5 |
| Beet pulp | 1.7 |
| Soybean meal | 2.6 |
| Corn grain | 6.3 |
| Premix^2^ | 0.7 |
| Chemical composition, % DM^3^ |  |
| DM | 46.6 |
| NEL, Mcal/kg | 1.34 |
| CP | 14.8 |
| NDF | 44.9 |
| ADF | 30.1 |
| CF | 3.2 |
| Starch | 17.8 |
| Ash | 9.8 |
| Ca | 0.70 |
| P | 0.44 |

^1^Dry distillers’ grains with solubles

^2^Premix contained (per kg of diet on DM basis): vitamin A 200,000 IU, vitamin D 70,000 IU, vitamin E 1,000 IU, Fe 2,000 mg, Cu 600 mg, Zn 2,400 mg, Mn 1,300 mg, I 6 mg, Co 7 mg

^3^Chemical composition was on dry matter (% DM) basis; CF: crude fat. NEL was predicted based on NRC (2001)
