# Supplementary material for: Elevated maternal non-esterified fatty acid concentrations during late gestation are associated with altered skeletal muscle development and mitochondrial dynamics related-markers in calves
Source: J Anim Sci Biotechnol. 2026 Jun 9;17:114. doi: 10.1186/s40104-026-01422-x (PMC13248329; doi:10.1186/s40104-026-01422-x)
Supplement: Supplementary file 2 — Additional file 2: Table S2. Primer sequences used in qPCR analyses. [file 40104_2026_1422_MOESM2_ESM.docx]

**Table S2** Primers used in qPCR analyses

| **Gene** | **Primers (5’→3’)** | **Amplicon length, bp** |
| --- | --- | --- |
| ***Bos*** |  |  |
| *DRP1* | Forward: GGAAGGAGGCTGACTCTGGG | 192 |
|  | Reverse: TCTTTCCACTGCTCTGCGTT |  |
| *FIS1* | Forward: TCTGTGGAGGACCTGCTGAAAT | 118 |
|  | Reverse: CGTTGTACTTGCTTCGCACC |  |
| *BAX* | Forward: GACAGGGGCCCTTTTGCTTC | 188 |
|  | Reverse: CCACAGCTGCGATCATCCTCT |  |
| *MFN1* | Forward: ACAGCACATGGAAAGATGCC | 132 |
|  | Reverse: CCCTGTGCTTTGTGCTTTCT |  |
| *MFN2* | Forward: AGAGGGCTCAGAGGAGAAGA | 133 |
|  | Reverse: CTTGAGAAGCGGACACTTGG |  |
| *NRF1* | Forward: TCACCATGGCACTCAACAGG | 107 |
|  | Reverse: GTGACAAAGTGAGGTACGACG |  |
| *NRF2* | Forward: CCACCAGTACCGCCGTGTC | 153 |
|  | Reverse: ATGTCAATCAAATCCATGTCCTGCT |  |
| *RELA* | Forward: CTCATCTTCCCTGCAGAGCC  Reverse: ATAGTGGGGTGGGTCTTGGT | 164 |
| *18S rRNA* | Forward: GTAACCCGTTGAACCCCATT | 98 |
|  | Reverse: CCATCCAATCGGTAGTAGCG |  |
| *GAPDH* | Forward: GGCGTGAACCACGAGAAGTATAA | 119 |
|  | Reverse: CCCTCCACGATGCCAAAGT |  |
| ***Mus*** |  |  |
| *Myh1* | Forward: GAATGGCAAGACGGTGACTGTG | 142 |
|  | Reverse: GGAAGCGTAGCGCTCCTTGAG |  |
| *Myh2* | Forward: ATCAACCAGCAGCTGGACACCA | 164 |
|  | Reverse: TCCAGCACGAACATGTGGTGGT |  |
| *Myh4* | Forward: CCAATGAAACCAAGACTCCTGG | 192 |
|  | Reverse: TGCTATCGATGAACTGTCCCTC |  |
| *Nrf1* | Forward: CCGTGTCCCAGAAAGCTTGG | 83 |
|  | Reverse: TTTCCAGAGAACCCCGAGTC |  |
| *Nfe2l1* | Forward: ACTACAGTCCCAGCAGAGTGAT | 144 |
|  | Reverse: AGACACTGCACTGCAACAAG |  |
| *Dnm1l* | Forward: CCAGAGGAACTGGTGTGGTC | 137 |
|  | Reverse: ACCCCATTCTTCTGCTTCAACT |  |
| *Fis1* | Forward: GACCCTAGCGTGCTTTCTGT | 326 |
|  | Reverse: CCTTTGGGCAACAGCTCCTT |  |
| *Nfkb1* | Forward: GCAGGAACTCAAGGGAGCTAAG | 123 |
|  | Reverse: TCTTTAACAGACCGCACAGC |  |
| *Rn18s* | Forward: GTAACCCGTTGAACCCCATT | 98 |
|  | Reverse: CCATCCAATCGGTAGTAGCG |  |
| *Gapdh* | Forward: GGCGTGAACCACGAGAAGTATAA | 119 |
|  | Reverse: CCCTCCACGATGCCAAAGT |  |

*DRP1*, Dynamin-Related Protein 1; *FIS1*, Mitochondrial Fission 1 Protein; *BAX*, BCL-2-Associated X Protein; *MFN1*, Mitofusin 1; *MFN2*, Mitofusin 2; *NRF1*, Nuclear Respiratory Factor 1; *NRF2*, Nuclear Factor Erythroid 2-Related Factor 2; *RELA*, v-rel Avian Reticuloendotheliosis Viral Oncogene Homolog A; *Myh1*, Myosin Heavy Chain 1; *Myh2*, Myosin Heavy Chain 2; *Myh4*, Myosin Heavy Chain 4; *Nrf1*, Nuclear Respiratory Factor 1; *Nfe2l1*, Nuclear Factor Erythroid 2-Like 1; *Dnm1l*, Dynamin 1-Like; *Fis1*, Mitochondrial Fission 1 Protein; *Nfkb1*, Nuclear Factor Kappa B Subunit 1; *18S rRNA /* *Rn18s*, 18S Ribosomal RNA; *GAPDH /* *Gapdh*, Glyceraldehyde-3-Phosphate Dehydrogenasee
