# Supplementary figures and images for: Elevated maternal non-esterified fatty acid concentrations during late gestation are associated with altered skeletal muscle development and mitochondrial dynamics related-markers in calves
Source: J Anim Sci Biotechnol. 2026 Jun 9;17:114. doi: 10.1186/s40104-026-01422-x (PMC13248329; doi:10.1186/s40104-026-01422-x)

# Supplementary file

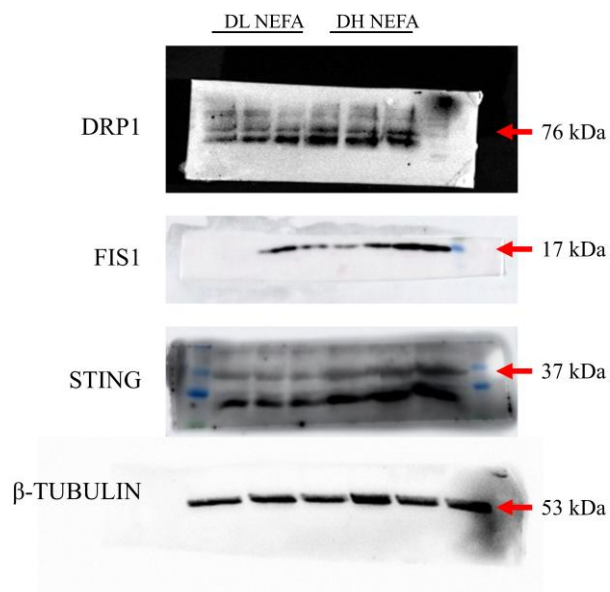

Original blots in Fig. 5A

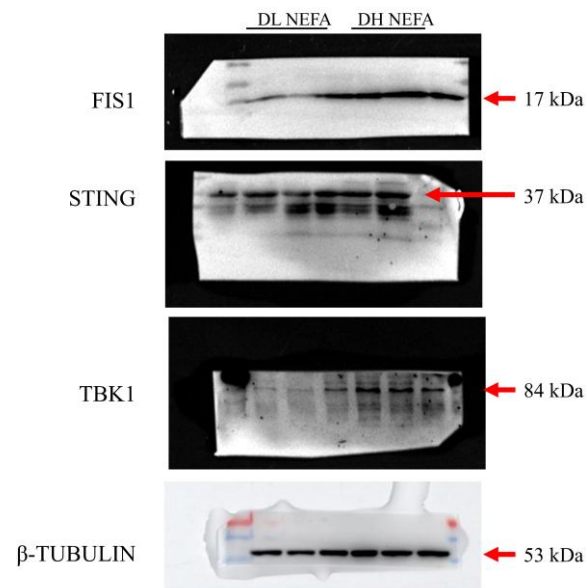

Original blots in Fig. 8A

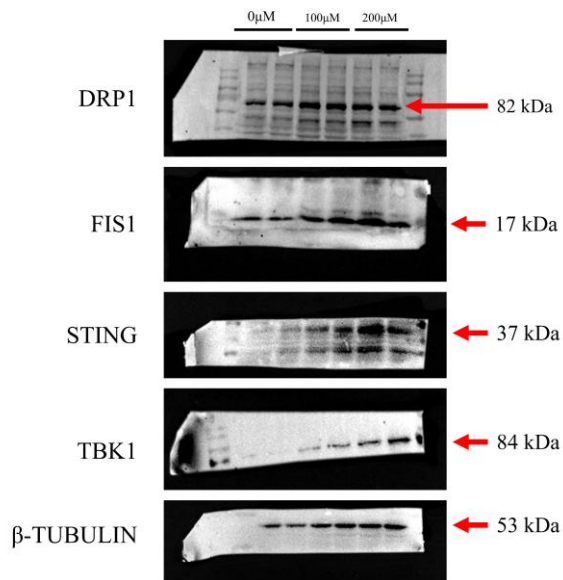

Original blots in Fig. 9D

Supplement: Supplementary file 3 — Additional file 3. The original Western blot images [file 40104_2026_1422_MOESM3_ESM.pdf]
